# Predictive and reactive changes in antioxidant defence system in a heterothermic rodent

**DOI:** 10.1007/s00360-020-01280-7

**Published:** 2020-05-20

**Authors:** Małgorzata Jefimow, Anna S. Przybylska-Piech, Michał S. Wojciechowski

**Affiliations:** 1 Department of Animal Physiology and Neurobiology, Faculty of Biological and Veterinary Sciences, Nicolaus Copernicus University, ul. Lwowska 1, 87-100 Toruń, Poland; 2 Department of Vertebrate Zoology and Ecology, Faculty of Biological and Veterinary Sciences, Nicolaus Copernicus University, ul. Lwowska 1, 87-100 Toruń, Poland

**Keywords:** Oxidative stress, Antioxidant defence, Non-shivering thermogenesis, Winter phenotype, Polymorphism, Photoresponsiveness, Heat production, Seasonal adjustments

## Abstract

Living in a seasonal environment requires periodic changes in animal physiology, morphology and behaviour. Winter phenotype of small mammals living in Temperate and Boreal Zones may differ considerably from summer one in multiple traits that enhance energy conservation or diminish energy loss. However, there is a considerable variation in the development of winter phenotype among individuals in a population and some, representing the non-responding phenotype (non-responders), are insensitive to shortening days and maintain summer phenotype throughout a year. Differences in energy management associated with the development of different winter phenotypes should be accompanied by changes in antioxidant defence capacity, leading to effective protection against oxidative stress resulting from increased heat production in winter. To test it, we analysed correlation of winter phenotypes of Siberian hamsters (*Phodopus sungorus*) with facultative non-shivering thermogenesis capacity (NST) and oxidative status. We found that in both phenotypes acclimation to winter-like conditions increased NST capacity and improved antioxidant defence resulting in lower oxidative stress (OS) than in summer, and females had always lower OS than males. Although NST capacity did not correlate with the intensity of OS, shortly after NST induction responders had lower OS than non-responders suggesting more effective mechanisms protecting from detrimental effects of reactive oxygen metabolites generated during rewarming from torpor. We suggest that seasonal increase in antioxidant defence is programmed endogenously to predictively prevent oxidative stress in winter. At the same time reactive upregulation of antioxidant defence protects against reactive oxygen species generated during NST itself. It suggests that evolution of winter phenotype with potentially harmful characteristics was counterbalanced by the development of protective mechanisms allowing for the maintenance of phenotypic adjustments to seasonally changing environment.

## Introduction

Living in a seasonal environment requires periodic changes in animal phenotype which occur at the physiological, morphological and behavioural levels. Phenotype, as a group of individuals of similar characters, may be understood as a product of natural selection favouring traits allowing for the best possible adjustment to the environment. When photoperiod shortens and ambient temperature (*T*_a_) decreases, small endotherms living in Temperate and Boreal Zones develop winter phenotype that may differ considerably from summer one in multiple traits that enhance energy conservation or diminish energy loss. Winter phenotype includes changes in body mass (*m*_b_), fur properties, metabolic heat production, reproductive traits, behaviour, torpor use (in heterothermic species), and body temperature (Lovegrove 2005). However, not all individuals within populations respond to changes in photoperiod in the same way. Those which represent the non-responding phenotype (non-responders) are insensitive to shortening days (Nelson 1987; Prendergast et al. 2001; Boratyński et al. 2016, 2017; Przybylska et al. 2019a, b). Individuals responding to short photoperiod (responders) show all traits of the winter phenotype, non-responders maintain their summer phenotype also throughout winter, and partial-responders may develop only some traits characteristic of the winter phenotype (Heldmaier and Steinlechner 1981b; Nelson 1987; Moffat et al. 1993; Ruf et al. 1993; Karebeshian et al. 1994; Broussard et al. 2009; Kaseloo et al. 2014; Przybylska et al. 2017, 2019a, b). In winter responders, contrary to non-responders, regress gonads and cease reproduction (Nelson 1987; Broussard et al. 2009; Kaseloo et al. 2012, 2014), decrease *m*_b_ (Hoffman 1973, Moffat et al. 1993, Bernardet al. 1997), change fur colour and its properties (Freeman and Goldman 1997a, b; Goldman et al. 2000), and in case of heterothermic species use torpor (Hoffmann 1973; Heldmaier and Steinlechner, 1981a, b; Lynch and Puchalski 1986). This polymorphism of winter phenotype is observed in many rodents, and the proportion of non-responding individuals depends on species, reaching up to 80% in prairie voles *Microtus ochrogaster* (Nelson 1985), 50% in white footed mice *Peromyscus leucopus* (Whitaker 1949), 47% in Turkish hamsters *Mesocricetus brandti* (Ogilvie and Stetson 1990), and 25% in deer mice *Peromyscus maniculatus* (Desjardins and Lopez 1983). Responders and non-responders may differ in the characteristic of circadian rhythm (Puchalski and Lynch 1988; Kliman and Lynch 1991; Gorman and Zucker 1997), melatonin synthesis (Puchalski and Lynch 1986), and the activity of suprachiasmatic nuclei neurons (Margraff et al. 1991). Non-responders are less active than responders and their nocturnal activity is shorter (Puchalski and Lynch 1986). Photoresponsiveness does not correlate with sex (Puchalski and Lynch 1991) but is heritable (Lynch et al. 1989; Goldman and Goldman 2003). Despite numerous studies (Puchalski and Lynch 1986, 1988; Lynch et al. 1989; Kliman and Lynch 1992; Bernard et al. 1997, Freeman and Goldman 1997a, b; Freeman and Goldman 1997a; Gorman and Zucker 1997; Anchordoquy and Lynch 2000; Goldman et al. 2000; Goldman and Goldman 2003; Diedrich et al. 2015), the phenomenon of polymorphism in winter phenotype still remains unexplained, although it may affect fitness,  altering both longevity (Turbill et al. 2012) and reproduction (Place et al. 2004; Heideman et al. 2005; Place and Cruickshank 2009; Przybylska et al. 2019b).

The existence of polymorphism of winter phenotype brings about a great opportunity to study costs and benefits of developing different morphological and physiological traits which affect survival in stochastic environments. In our outbred colony of Siberian hamsters *Phodopus sungorus* the proportion of non-responders varies between 50 and 75%. In winter, or after acclimation to winter-like conditions, responding Siberian hamsters enter daily torpor and reduce *m*_b_, show gonadal regression and change fur from grey to white. Non-responders do not show above changes, while partial responders show only some traits of winter phenotype (Przybylska et al. 2019a, b). As in other small placental mammals, also in Siberian hamsters non-shivering thermogenesis (NST) is the primary source of heat (Janský 1973; Gaudry and Campbell 2017), and its capacity increases seasonally in both phenotypes (Puchalski and Lynch 1986). Thermogenic capacity of brown adipose tissue (BAT), the main organ for NST, is species-specific and depends on environmental conditions; it increases when photoperiod shortens and *T*_a_ decreases (Heldmaier et al. 1990; Ruf et al. 1993). Exposure to short photoperiod alone is often sufficient to increase NST capacity, although low *T*_a_ improves it further (Heldmaier and Buchberger 1985; Rafael et al. 1985a, b; Wiesinger et al. 1989). Numerous studies showed that winter, or acclimation to short photoperiod (SP) or cold, or both enhance BAT-mediated NST (Lynch 1973; Heldmaier et al. 1982, 1985; Klaus et al. 1988; Wiesinger et al. 1990; Merritt et al. 2001; Jefimow et al. 2004a, b; Zhao et al. 2010; Oelkrug et al. 2013, 2014). The mechanism of NST is based on the uncoupling of cellular respiration from ATP synthesis thanks to the presence of uncoupling protein 1 (UCP1) located in the inner mitochondrial membrane (Cannon and Nedergaard 2004). Because NST in BAT requires intense oxygen consumption, it led to the conclusion that BAT activity induces oxidative stress (DeQuiroga 1992). Oxidative stress is defined as an imbalance between production of reactive oxygen species (ROS) and antioxidant defence which may result from increased production of ROS or diminished levels of antioxidants, or both (Halliwell and Whiteman 2004). Indeed, the production of ROS in BAT mitochondria is very high, both in the coupled and the uncoupled states (Mailloux et al. 2012; Schönfeld and Wojtczak 2012; Adjeitey et al. 2013). The majority of reactive oxygen species are generated in mitochondria as a consequence of oxidative phosphorylation at complexes I and III in the electron transport chain (Brand, 2000; Nicholls 2004; Balaban et al. 2005). However, the results of experimental studies asking about the involvement of UCP1 in ROS regulation are equivocal (Casteilla et al. 2001; Shabalina et al. 2014; Jastroch 2017). On the one hand, activation of BAT thermogenesis induces an increase in ROS concentration (Mailloux et al. 2012; Schönfeld and Wojtczak 2012; Chouchani et al. 2016). On the other hand, there is evidence that UCP1 activation decreases ROS production (Oelkrug et al. 2010; Stier et al. 2014) by lowering the potential of the inner mitochondrial membrane (Echtay et al. 2002a, b; Mookerjee et al. 2010), and thus it is considered to possess antioxidant properties and prevent oxidative stress (Dlasková et al. 2010; Oelkrug et al. 2014). Moreover, ROS can be a direct activator of UCP1 (Echtay et al. 2002a, b) and support UCP1-mediated thermogenesis (for review see Chouchani et al. 2017). It was even suggested that UCP1 evolved as a mechanism reducing mitochondrial ROS production, not as a thermogenic one (Clarke and Porter 2012; Oelkrug et al. 2010, 2014). Antioxidant properties of UCP1 (Oelkrug et al. 2010, 2013, 2014) as well as activity of antioxidant enzymes in BAT (DeQuiroga 1992) increase after acclimation to cold. Therefore, seasonal increase in thermogenic capacity of BAT, resulting from the increase in UCP1 content, correlates with the effectiveness of antioxidant defence (Oelkrug 2010; Zhou et al. 2015). The antioxidant role of UCP1 was further supported by the studies of UCP1-knockout mice, in which a cold-induced increase in shivering thermogenesis led to much higher oxidative stress than in control animals which relied on non-shivering heat production (Stier et al. 2014).

Undoubtedly, seasonal increase in NST capacity facilitates maintenance of high body temperature in the cold. In heterothermic mammals, like Siberian hamsters, NST plays a key role during rewarming from torpor, supplying a large amount of heat before shivering thermogenesis can be brought into action (Lyman and Chatfield 1950). We found that the seasonal increase of NST capacity correlates temporarily with the increase in frequency of torpor episodes (Jefimow et al. 2004a, b). Since torpor is used only by responders and because its use correlated with NST capacity, we assumed that the magnitude of heat production by NST and its seasonal changes differ between phenotypes. This in turn would lead to significant differences in oxidative stress between responders and non-responders.

We asked three questions: 1/how does oxidative status (pro- and antioxidative arms of oxidative balance) of different winter phenotypes change seasonally, 2/does photoresponsiveness correlate with development of NST capacity; namely, whether non-responders which do not enter daily torpor have lower NST capacity than responders, and 3/do changes, or differences in NST capacity, correlate with changes or differences in oxidative status of animals? By answering these questions we tested whether a seasonal increase in NST capacity is paralleled by an increase in antioxidant capacity, which protects against oxidative stress that might arise from increased heat production in winter. We predicted that seasonal changes in antioxidant capacity differ between phenotypes, being highest in the responding phenotype, and lowest in the non-responding one. To answer the first question we measured total oxidative status of Siberian hamsters (*Phodopus sungorus*) acclimated to summer-like and then to winter-like conditions. In these experiments we also measured basal metabolic rate (BMR) to test if high metabolic rate correlates with higher ROS production (Harman 1956; Sohal et al. 2002; Speakman et al. 2002, 2004; Mookerjee et al. 2010; but see Costantini et al. 2010). To answer the latter two questions we measured seasonal changes in NST capacity and analysed its correlation with oxidative status in winter acclimated Siberian hamsters representing responding and non-responding phenotypes. We also measured oxidative status immediately after NST induction by injection of exogenous noradrenaline (NA).

## Material and methods

### Animals and housing

This study was done at the Nicolaus Copernicus University in Toruń, Poland. All experimental procedures were approved by the Local Committee for Ethics in Animal Research in Bydgoszcz, Poland (decision numbers 3/2015, 31-33/2015, 35/2015, 30/2016). We used 160 adult Siberian hamsters (*Phodopus sungorus*, 80 males and 80 females) originating from our breeding colony which were born under 16 h photoperiod (16L: 8D, lights on at 05:30) and *T*_a_ = 20 ± 2 °C. The colony consists of animals descending from hamsters captured in the wild, which we obtained from Dr. Dietmar Weinert from University of Halle-Wittenberg, Germany and from Prof. Gerhard Heldmaier from Philipps University of Marburg, Germany. Animals were maintained under the same conditions for three months (summer-like, or summer acclimation). Then all hamsters were acclimated to winter-like conditions (winter; *T*_a_ = 10 °C, LD 8:16, lights on at 08:30) for 4 months. Throughout the study hamsters were housed individually in standard laboratory cages (33 × 20 × 18 cm; cage model 1246; Tecniplast, Italy) with sawdust as bedding material, and food (standard rodent diet; Labofeed B, Morawski, Kcynia, Poland) and water available ad libitum.

Every 2 weeks hamsters were weighed to ± 0.1 g with an electronic balance (SPU402; Ohaus, USA) to monitor *m*_b_ changes during the course of acclimation. Body mass was also measured before and after each respirometry trial.

### Defining hamster phenotype

**Photo 1:**
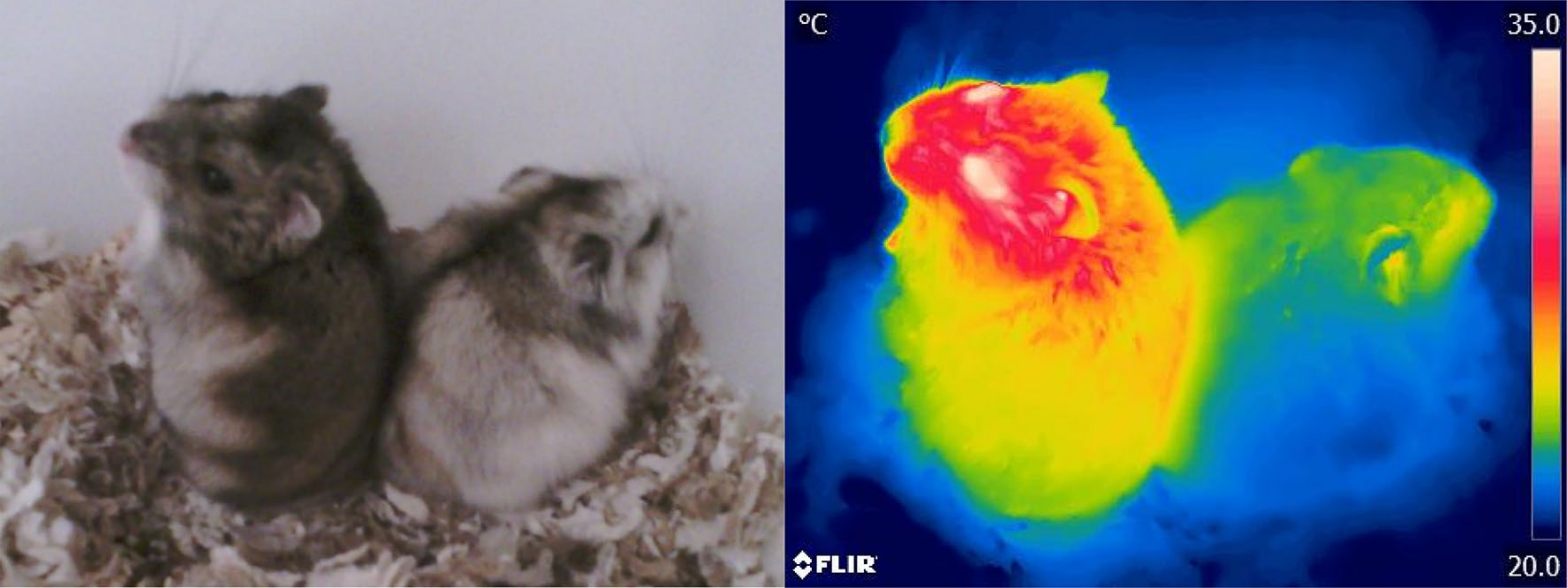
Different winter phenotypes frequently occur in the same litter. These photographs, taken with thermal camera (FLIR T540), show siblings from our colony kept under SP and *T*_a_ of 20 °C, one of which is non-responder (grey fur, on the left) and the other is responder (whitening, on the right). While taking photographs, non-responder was active while responder was torpid

We classified individuals as responders (R), non-responders (NR), or partial-responders (PR) based on both torpor use (subcutaneous temperature < 32 °C) and fur colour (Photo 1). Namely, responders entered daily torpor and turned white, while non-responders did not enter daily torpor and remained grey. After acclimation to winter 33 out of 160 hamsters were classified as responders, 98 as non-responders and 22 as partial-responders. Individuals classified as partial-responders entered daily torpor but remained grey, or moulted to white fur but did not enter torpor, and increased, decreased or did not change *m*_b_. They were excluded from further analyses because of high heterogeneity of this group. Seasonal adjustments in different traits of hamster physiology, morphology and behaviour have different physiological and genetic underpinnings (for a review see: Cubuk et al. 2016; Williams et al. 2017; Dardente et al. 2019), hence any unequivocal interpretation of changes observed in this heterogenous group would be hindered. Because we hypothesized that oxidative status (pro-and antioxidative markers) is related to torpor use and that NST capacity is related to winter phenotype, we are convinced that excluding partial responders from analyses allowed us to avoid misinterpretation of obtained results.

Subcutaneous temperature was measured with miniature data loggers (model TL3-1-27, mass 0.8 g, accuracy of 0.3 °C from 0 to 45 °C; constructed by Dr. Dmitry Petrovsky from Russian Academy of Sciences, Novosibirsk, Russia) which were implanted into the interscapular region before acclimation to winter under ketamine (40 mg kg^−1^; Ketamina 10%, Biowet, Puławy, Poland) and xylazine (8 mg kg^−1^; Sedazin 2%, Biowet, Puławy, Poland) anaesthesia. Before implantation all loggers were coated in paraffin wax and calibrated against a precise mercury-in-glass thermometer to the nearest 0.5 °C. Data obtained from the loggers were used only to differentiate phenotypes.

### Basal metabolic rate and non-shivering thermogenesis

If an animal was found torpid on the day of metabolic trial, we postponed the measurement to the next day. Hamster metabolic rate was quantified by indirect calorimetry using open flow respirometry. Basal metabolic rate was measured at *T*_a_ of 28.5 °C which is within thermoneutral zone of Siberian hamster (Gutowski et al. 2011). Measurements commenced at 09:00 h, i.e. during animals' ρ-phase and lasted for ~ 7 h. We used two parallel respirometry systems that allowed to measure gas exchange in 14 hamsters at once. The system was set up so that respiratory gas exchanges of two animals were measured simultaneously. Measurements were taken for 5 min with reference air being sampled twice for 4 min between animals. Thus, gas exchange was measured in each hamster every 44 min throughout the experiment. Outside air was pulled from outside the building using air pump and compressed in a balloon, then dried and scrubbed of CO_2_ with a PureGas Generator (Puregas, Westminster, CO, USA). Next, air was continuously pushed through 14 air-tight 0.85 L respirometry chambers constructed of polypropylene containers (HPL 808, Lock&Lock, Hana Cobi, South Korea) with a constant flow rate of ~ 330 mL min^−1^ per chamber. With this flow rate CO_2_ concentration in excurrent air did not exceed 0.25% during the steady-state measurement of gas exchange. All chambers were placed in a temperature-controlled cabinet (ST-1200, Pol-Eko-Aparatura, Wodzisław Śląski, Poland). Airflow was regulated upstream of chambers using precise needle valves. Excurrent flow was selected sequentially with a computer-controlled multiplexer (MUX, Sable Systems Int., USA) and flow rate though a selected chamber was measured downstream using a mass flow meter (FlowBar-8, Sable Systems Int., USA). A multiplexer was set up so that two parallel, separated air streams leading to two gas analysers were selected at the same time. After flow measurement, air from each gas stream was subsampled at a rate of ~ 100 mL min^−1^ and water vapour pressure in the subsampled air was measured with water vapour analysers (RH-300, Sable Systems Int., USA). This was later used to correct air flow rate for the water vapour content (Lighton 2008). Then air was dried in a column of magnesium perchlorate (Sigma-Aldrich, USA), and subsequently excurrent fractional concentrations of CO_2_ (FeCO_2_) and O_2_ (FeO_2_) were measured using a FoxBox-C integrated CO_2_ and O_2_ analyser, or with a FC-10a O_2_ analyser (Sable Systems Int., USA) and CA10 CO_2_ analyser (Sable Systems Int., USA). Rate of O_2_ consumption ($\dot{V}O_{2}$ mL O_2_ min^−1^) was calculated using Eq. 11.7 of Lighton (2008).

Non-shivering thermogenesis capacity was measured as an increase in metabolism after noradrenaline (NA) injection in non-anesthetized animals. We measured NST capacity in all summer- and winter-acclimated hamsters. Additionally, in 19 hamsters we also measured heat production after saline injection (in the same volume as NA) to infer the effect of stress of injection. Following Heldmaier and Buchberger (1985), to prevent severe hyperthermia, NST measurements were done at *T*_a_ = 25 °C, which is at the lower limit of thermoneutrality of winter-acclimated Siberian hamsters (Gutowski et al. 2011). After 40 min recording of resting gas exchange, NA (Levonor 1 mg mL^−1^, Polfa, Poland) was injected subcutaneously at a dose of 2.54 *m*_b_ (g)^−0.4^ (mg kg^−1^; Wunder and Gettinger 1996) and respiratory gas exchange was measured for the next 30 min. In each hamster, NST capacity was calculated as the difference between basal metabolic rate (BMR) and maximum metabolic rate after NA injection (average of the continuous maximum MR over 3 min). During NST measurements we used the respirometry system in which $\dot{V}O_{2}$ was measured in three animals at the same time. The flow rate of air was set at ~ 700 mL min^−1^ to prevent accumulation of CO_2_ in the chamber (the peak fractional concentration of CO_2_ in the excurrent air after NA-injection did not exceed 0.006). Flow rate was regulated with a needle valve and measured upstream of the respirometry chambers with a mass flow meter (FlowBar-8, Sable Systems Int., USA). A subsample of the excurrent air flow was taken at a rate of ~ 100 mL min^−1^ and dried in a column of magnesium perchlorate (Sigma-Aldrich, USA). In two animals FeCO_2_ and FeO_2_ were measured as above, while in one animal we measured only FeO_2_ with FC-10a O_2_ analyser (Sable Systems Int., USA). When both FeCO_2_ and FeO_2_ were known $\dot{V}O_{2}$ during the NST measurements was calculated using Eq. 10.6 of Lighton (2008). When only FeO_2_ was measured we used Eq. 10.2 (Lighton 2008) assuming respiratory exchange rate (RER) = 0.8 (Koteja 1996; this approach results in the smallest calculation error when FeCO_2_ is unknown.)

Metabolic rate was calculated in Watt’s (W) assuming respiratory exchange ratio (RER, $\dot{V}CO_{2} /\dot{V}O_{2}$) calculated from observed $\dot{V}CO_{2}$ and $\dot{V}O_{2}$ using oxyjoule equivalent after Lighton et al. (1987):

$$\normalsize MR(W) = \frac{{\dot{V}O_{2} (16 + 5.164RER)}}{60}$$

All elements of the respirometry system were controlled with a PC computer via an analog-to-digital interface (UI2, Sable Systems Int., USA) and data were acquired using ExpeData software (Sable Systems Int.) at 0.5 Hz.

### Oxidative status measurements

After acclimation to summer- and winter-like conditions, we measured markers of oxidative status in plasma, namely concentrations of reactive oxygen metabolites (ROM) and the biological antioxidant potential (BAP). This allowed us to study seasonal changes in oxidative status and its correlation with changes in physiology (BMR, NST, torpor use) within the same individuals.

Blood (~ 100 μL) was drawn from hamster retro-orbital sinus with heparinized hematocrit capillaries. After blood collection, a drop of analgesic 0.5% Alcaine solution (Proxymetacaini hydrochloridum; Alcon, Belgium) was applied on the eye. Blood was centrifuged at 6000 rpm and 20 μL of plasma was used for measurements. We used dROM-kits and BAP-kits of the Free Radical Analytical System (FRAS4 evolvo; H&D srl, Parma, Italy) following the manufacturer’s instructions. With dROM kits we measured total hydroperoxide in plasma (ROM, mg H_2_O_2_ dL^−1^) that comes from peroxidation of amino acids, lipids and proteins, representing free radicals from which ROM are formed, and may be released from intracellular compartment to blood (Ames et al. 1993). ROM are markers of early oxidative damage, which include primarily hydroperoxides (Costantini et al. 2007). With BAP-kits we measured the concentration of non-enzymatic plasma antioxidants (BAP, mmol vitamin C L^−1^). Both methods have been used previously in other studies on oxidative stress in vertebrates (Costantini et al. 2006, 2007; Schneeberger et al. 2013, 2014). Finally, oxidative stress (OS) index was calculated as ROM to BAP ratio (Costantini et al. 2006).

ROM and BAP were measured in 160 summer- and winter-acclimated hamsters and in 42 winter-acclimated hamsters immediately after NST measurements. In the latter case, blood was sampled immediately after calorimetry trials, i.e. 30 min after NA injection. Nineteen out of those 42 hamsters were also injected with saline, to control for the effect of injection stress, and blood was collected also 30 min after injection.

### Data analysis

To infer seasonal changes in *m*_b_, oxidative status (ROM, BAP and OS), BMR and NST (total and mass-specific, NST/*m*_b_), we used linear mixed effects models with animal ID set as a random factor to control for repeated measurements of individuals. In all analyses season, sex and phenotype were set as fixed factors. In models analysing seasonal changes in BMR and total NST, *m*_b_ was included as a covariate.

In analyses of correlation between NST and oxidative status (ROM, BAP, OS) measured after NA injection we used general linear models with season, sex and phenotype included as fixed factors and residuals from the relationship between maximum MR after NA-injection and *m*_b_ (residual NST) as a covariate.

Initial maximal models including all fixed factors and their possible interactions were simplified by stepwise elimination of insignificant terms (Crawley 2009). Assumptions of the linear modelling were checked post hoc by inspecting the distribution of residuals obtained from models (Grafen and Hails 2002).

Parameters of oxidative status after NA or saline injections were compared with paired* t*-test.

All statistical analyses were done in IBM SPSS v. 25 (IBM Corp., Armonk, NY, USA). Data are presented as estimated marginal means ± SE and statistical significance was accepted at *p* < 0.05.

## Results

### Seasonal changes of body mass in responding and non-responding hamsters

**Fig. 1:**
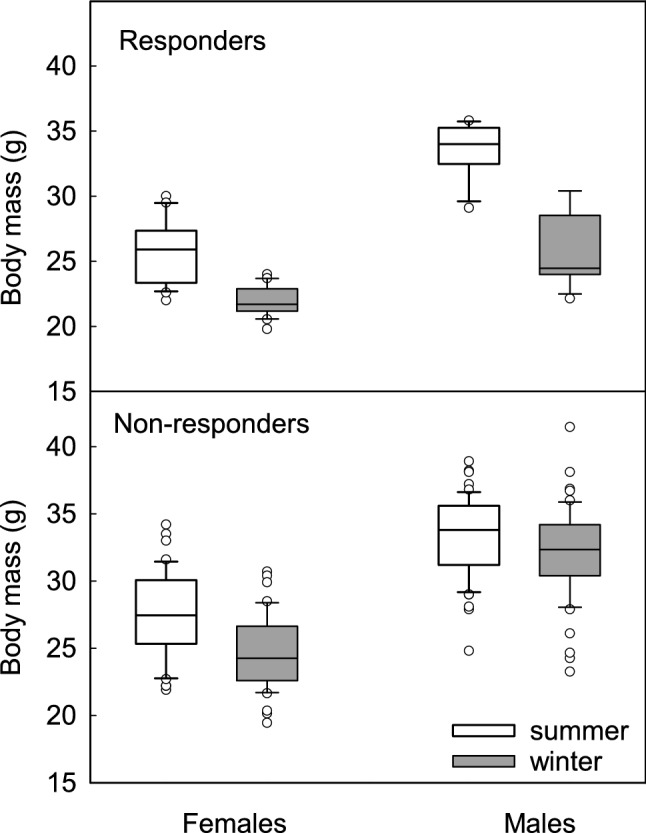
Body mass (g) of responding and nonresponding Siberian hamsters in summer (white boxes) and winter (grey boxes). Box indicates 25th and 75th percentiles, solid line stands for median and dots are outliers

Hamster *m*_b_ was lower in winter than in summer and this difference was affected by phenotype and sex (sex × season × phenotype: *F*(1, 127) = 18.50, *p* < 0.001; Fig. 1). In responding hamsters these differences were more pronounced than in non-responding ones, and in winter females were ~ 15% lighter than in summer while in males this difference was twice as much. On average, *m*_b_ of non-responding males did not differ seasonally (< 5% difference between summer and winter), while *m*_b_ of non-responding females was ~ 10% lower in winter than in summer.

### Effect of winter phenotype on seasonal changes in oxidative status

**Fig. 2:**
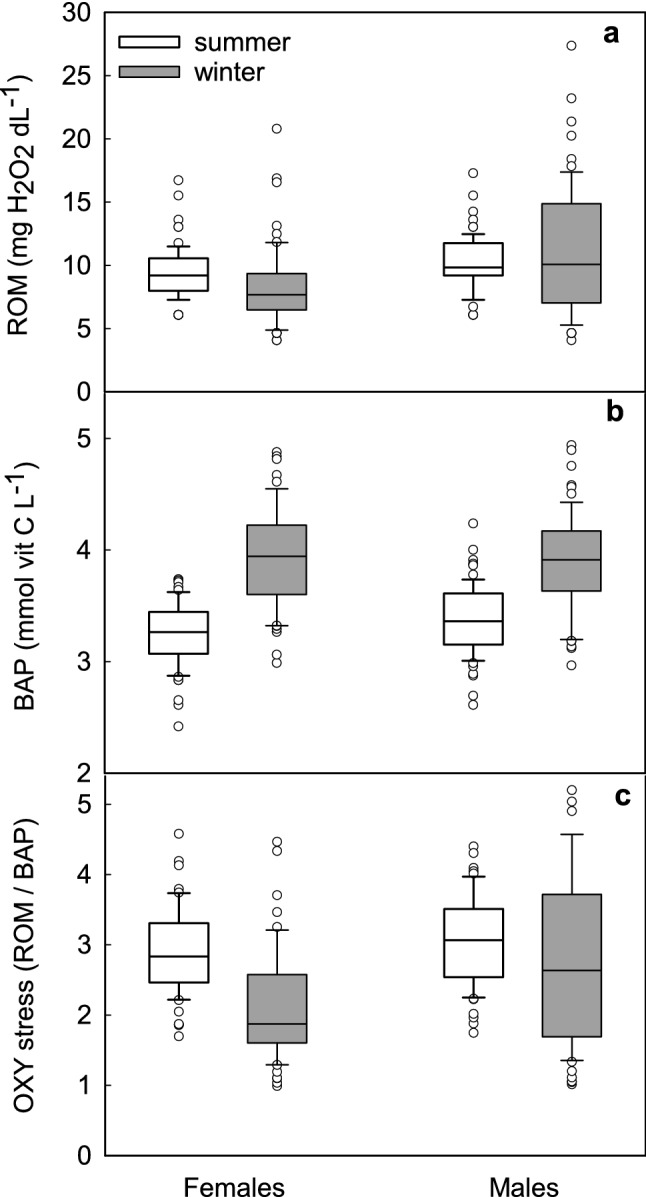
Reactive oxygen metabolites (ROM; mgH_2_O_2_ dL^−1^, **a**), biological antioxidant potential (BAP mmol vit C L^−1^, **b**) and oxidative stress (ROM/BAP, **c**) in summer- (white boxes) and winter-acclimated (grey boxes) male and female Siberian hamsters. Box indicates 25th and 75th percentiles, solid line stands for median and dots are outliers

Phenotypes did not differ in ROM [*F*(1, 127) = 0.052, *p* = 0.82] or BAP [*F*(1, 127) = 0.062, *p* = 0.804] concentrations. The ROM concentrations differed seasonally between males and females (sex × season interaction: *F*(1, 128) = 5.50, *p* = 0.021), namely male ROM did not differ seasonally, while females had lower ROM in winter (Fig. 2a). Moreover, in winter concentration of ROM in females was ~ 25% lower than in males, while in summer this difference was < 10%. BAP also was affected by sex and season (sex × season interaction: *F*(1, 128) = 4.773, *p* < 0.031). In summer BAP was significantly lower in females than in males, but this difference disappeared in winter because females increased BAP by ~ 18% (compared to summer) while males only by ~ 13% (Fig. 2b). In general there was no difference between phenotypes in oxidative stress [*F* (1, 127) = 0.123, *p* = 0.726]. However, OS was affected by sex and season [*F* (1, 128) = 6.665, *p* = 0.011], and OS in females in winter was lower than in summer as well as it was lower than OS in males in both seasons (Fig. 2c). Neither ROM nor BAP or OS correlated with residual BMR or with residual NST (*p* > 0.05).

### Seasonal changes in NST in responding and non-responding hamsters

**Fig. 3:**
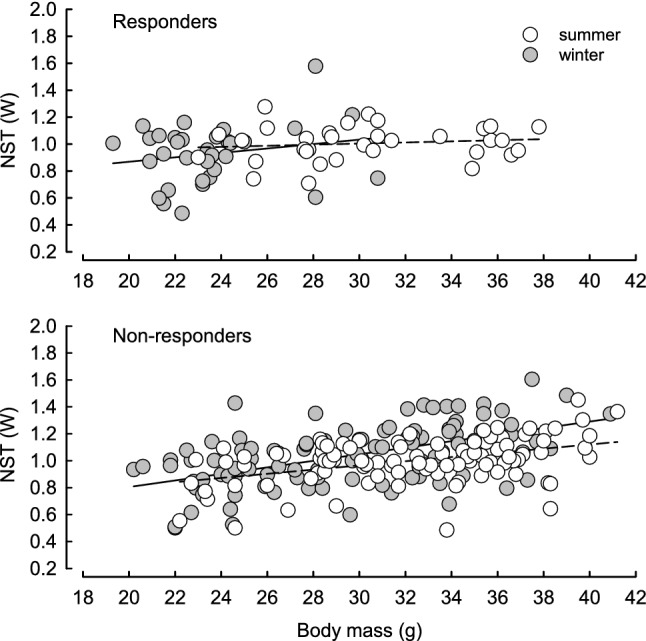
Relation between non-shivering thermogenesis capacity (NST, W) and body mass (g) in responding (upper panel) and non-responding (lower panel) Siberian hamsters acclimated to summer (white symbols, dashed regression lines) and winter conditions (grey symbols, solid regression lines)

Overall, NST correlated with *m*_b_ (*F* (1, 240.813) = 43.113, *p* < 0.001). However, this relationship differed between seasons [interaction season × *m*_b_: *F* (1, 165.332) = 5.636, *p* = 0.019; Fig. 3]. Irrespective of the phenotype, after adjusting for *m*_b_ NST in winter was ~ 8% greater than in summer.

**Fig. 4:**
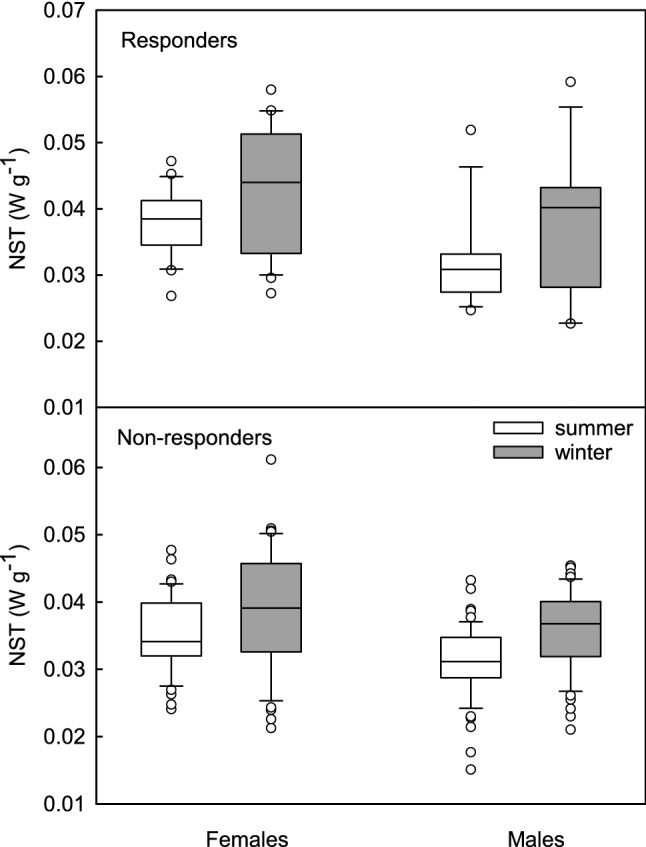
Mass-specific non-shivering thermogenesis (NST, W g^−1^) in summer- (white boxes) and winter-acclimated (grey boxes) male and female Siberian hamsters, non-responding and responding to short photoperiod. Box indicates 25th and 75th percentiles, solid line stands for median and dots are outliers

When calculated per unit of *m*_b_ (NST/mb; Fig. 4) NST correlated with season [*F*(1, 130.008) = 32.057, *p* < 0.001], phenotype [*F*(1, 128.009) = 6.755, *p* = 0.01] and sex [*F*(1, 128.009) = 27.906, *p* < 0.001]. NST/*m*_b_ was higher in winter than in summer and it was higher in responders than in non-responders, as well in females than in males. There was no interaction between fixed factors.

### Effect of winter phenotype on the correlation between NST and oxidative stress

**Fig. 5:**
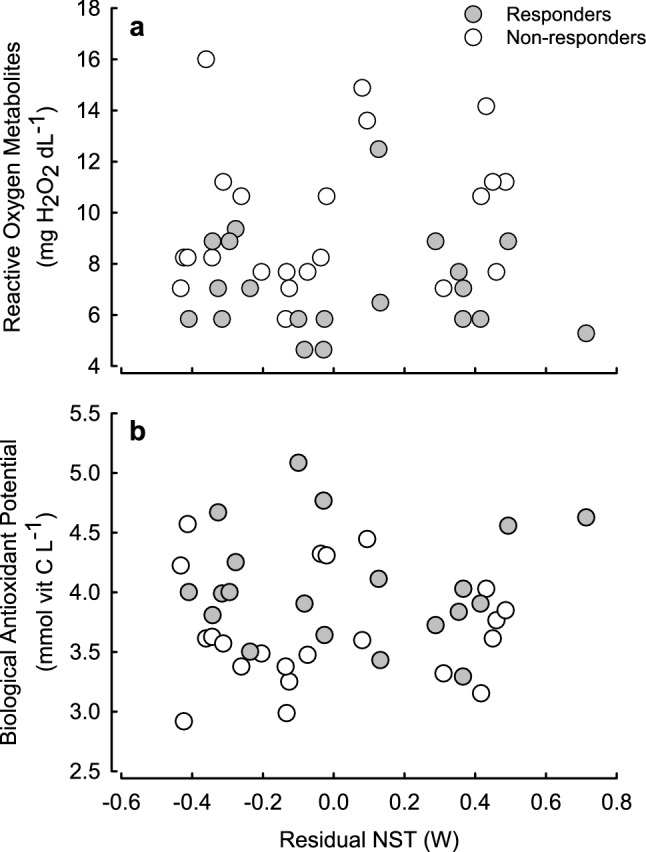
Relationship between Reactive Oxygen Metabolites (ROM, mg H_2_O_2_ dL^−1^; **a**), Biological Antioxidant Potential (BAP, mmol vit C L^−1^; **b**) and residual NST (W) in hamsters (*n* = 42) responding to seasonal changes in photoperiod (grey symbols) and not responding to seasonal changes (white symbols)

**Fig. 6:**
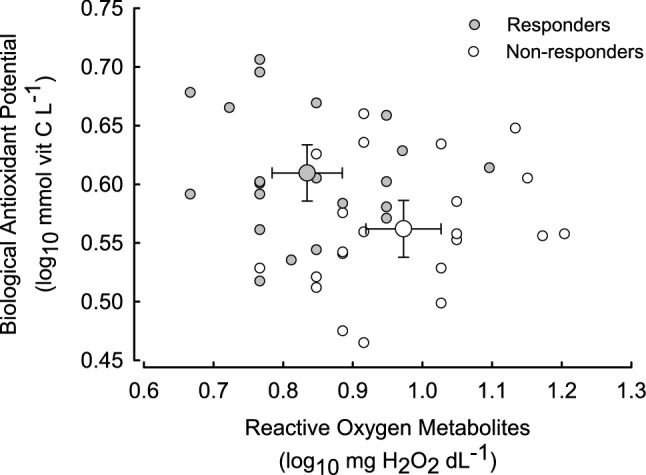
Reactive oxygen metabolites (ROM, mg H_2_O_2_ dL^−1^) and biological antioxidant potential (BAP, mmol vit C L^−1^) following NST induction with noradrenaline in Siberian hamsters (*n* = 42) responding (grey symbols) and non-responding (white symbols) to seasonal changes in photoperiod. Large points indicate mean and whiskers indicate 95% confidence interval of the mean

Measurements of oxidative status just after NA injection in winter (*n* = 42) showed that ROM concentration did not correlate with residual MR after NA-injection (Fig. 5), but correlated with phenotype [*F*(1, 40) = 12.060, *p* = 0.001] being higher in non-responders than in responders (9.8 ± 0.61 and 7.1 ± 0.44 mgH_2_O_2_ dL^−1^; respectively; *p* < 0.001; Fig. 6). Similarly, the concentration of BAP did not correlate with residual NST (Fig. 5). Yet, it differed between phenotypes [*F*(1, 40) = 6.840, *p* = 0.013, Fig. 6] and on average it was higher in responding than in non-responding individuals (4.1 ± 0.11 and 3.7 ± 0.10 mmol vitamin C L^−1^, respectively). As a consequence, OS after NA injection did not correlate with NST and depended on phenotype being lower in hamsters responding to seasonal changes in photoperiod [*F*(1, 40) = 19.692, *p* < 0.001].

**Fig. 7:**
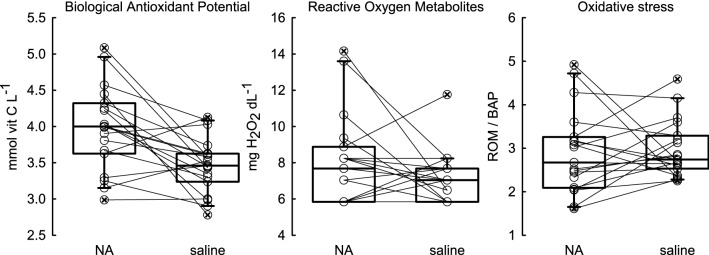
Biological antioxidant potential (BAP, mmol vit C L^−1^), reactive oxygen metabolites (ROM, mg H_2_O_2_ dL^−1^) and oxidative stress (ROM/BAP) after induction of facultative non-shivering thermogenesis (NA-injection) and control injection of saline solution which were measured in the same individuals of Siberian hamsters (*n* = 19)

ROM concentration after NA and saline injections did not differ (paired *t*-test: *t* = 1.254, *d.f.* = 18, *p* = 0.226). However, BAP was approximately 15% higher after NST induction than after saline injection (paired *t*-test: *t* = 3.553, *d.f.* = 18, = 0.002). This resulted in no difference in OS after NA or saline injections (paired *t*-test: *t *= − 0.287, *d.f.* = 18, *p* = 0.778; Fig. 7).

## Discussion

### Polymorphism of winter phenotype

In small Temperate-Zone mammals, winter phenotype differs considerably from summer one (Lovegrove 2005) but only in individuals responding to changes in day length. The most important exogenous signal driving seasonal response is photoperiod, while ambient temperature has minor effect (Ruf et al. 1993). In winter responding Siberian hamsters enter daily torpor and reduce body mass, show gonadal regression and change fur from grey to white. Non-responders do not show above changes, while partial responders show only some traits of winter phenotype (Hoffmann 1973; Heldmaier and Steinlechner 1981a, b; Przybylska et al. 2019a, b). In our study, 33 out of 160 hamsters were classified as responders, 98 as non-responders and 22 as partial-responders. It seems unlikely that photononresponsiveness resulted from long-term breeding under laboratory conditions, because non-responders appeared also among third-generation laboratory stock derived from wild–caught animals (Lynch et al. 1989; Kliman and Lynch 1992).

### Sex and phenotype differences in non-shivering thermogenesis capacity and oxidative stress

The results support our hypothesis that seasonal changes in thermoregulatory heat production are accompanied by changes in the antioxidant defence resulting in effective protection against detrimental consequences of oxidative stress. In winter oxidative stress in Siberian hamsters was lower than in summer (Fig. 2c) what could result from higher NST capacity and higher UCP1 content, known for its antioxidant properties (Oelkrug et al. 2010, 2013, 2014). Independent of season or phenotype, females had lower OS than males which presumably resulted from the protective action of oestrogens in summer (Persky et al. 2000; Borrás et al. 2003) and higher mass-specific capacity of NST in winter (Fig. 4). Responders did not differ from non-responders in oxidative stress, likely because they did not differ in *m*_b_ - adjusted BMR or *m*_b_- adjusted NST. Thus, the use of daily torpor itself did not induce marked increase in NST capacity, because both, responders and non-responders rely on non-shivering thermogenesis to cope with cold when active on a daily basis. Although the capacity for NST did not differ between phenotypes when adjusted for *m*_b_, mass-specific NST (NST/*m*_b_) in winter was higher in responders than in non-responders (Fig. 4). This indicates that the seasonal increase in NST capacity was partially a product of *m*_b_ changes. In our experiments hamsters of both phenotypes decreased *m*_b_ in winter by ~ 5–30%, depending on the phenotype and sex (Fig. 1). Having similar heat production, small-bodied responders gained more heat per unit of *m*_b_ than non-responders which did not reduce *m*_b_ in response to winter-acclimation. A similar pattern was observed in Puchalski and Lynch’s (1986) study. Reanalysis of their data indicates that in winter mass-specific NST capacity of responding hamsters increased by ~ 16% while *m*_b_ decreased by ~ 15% (Puchalski and Lynch 1986). As a consequence, total capacity for NST per animal did not change, yet the effectiveness of heat production increased.

### Reactive and predictive antioxidant defence

Our results imply two possible mechanisms of seasonal increase in antioxidant defence: *reactive defence*, in which increase in BAP is a consequence of higher ROS concentration due to higher heat production in winter, and *predictive defence*, where an increase in BAP results from seasonal changes in overall antioxidant capacity to prevent oxidative stress in winter. Lack of direct correlation between the magnitude of non-shivering heat production and oxidative stress points towards the latter option (Fig. 5). Despite differences in *m*_b_ between winter phenotypes, ROM or BAP concentrations did not correlate with *m*_b_. Inverse relationships between markers of oxidative status and *m*_b_ were reported in mice, but these were rather related to behavioural characteristics of animals than to *m*_b_ itself (Costantini et al. 2008). Because hamsters of different winter phenotypes did not differ in behavioural traits (Przybylska et al. 2019a), animal behaviour rather did not contribute to changes in oxidative status.

Our results also indicate that NST directly affects parameters of oxidative status. Although ROM concentrations in NA- and saline-injected animals did not differ, BAP was approximately 15% higher after NST induction than after saline injection (Fig. 7). Moreover, after NA injection responders had lower concentration of ROM in blood and greater BAP than non-responders (Fig. 6). These results indicate that ROS generated during facultative NST did not contribute to oxidative stress and also suggest that capacity for higher BAP, correlated with lower ROM concentration in responding hamsters might have protected them against higher susceptibility to OS while rewarming from torpor.

### Seasonal changes in antioxidant defence

The correlation between heterothermy and oxidative status was more extensively studied during hibernation than during daily torpor. Although daily and seasonal torpor differ markedly in depth and duration of particular episodes, animal body mass and energy savings (Ruf and Geiser 2015), yet the physiological mechanism of heat production during rewarming, namely NST is the same. Buzadźić et al. (1990a, b) suggested that high antioxidant defence in hibernators was a consequence of NST in BAT, especially during rewarming from hibernation torpor. An increased concentration of antioxidants during hibernation bout and also during rewarming phase would protect against oxidative stress during arousal, when metabolic rate dramatically increased (Buzadźić et al. 1990a, b; Tøien et al. 2001; Ma et al. 2005; Okamoto et al. 2006; Filho et al. 2007; Morin and Storey 2007; Astaeva and Klichkhanov 2009; Vucetic et al. 2013; Yin et al. 2016; but see Page et al. 2009). Although rewarming may increase the amount of carbonyl proteins and lipid peroxide end products in BAT, indicating oxidative stress in this tissue, nonetheless hibernation torpor itself did not induce oxidative stress (Orr et al. 2009). Undoubtedly, the above studies also showed that antioxidant defence is not fixed but changes seasonally (Buzadźić et al. 1992, 1997; Belló-Klein et al. 2000). The importance of a winter increase in antioxidant capacity may be also related to animal diet. In present study hamsters received the same diet during summer and winter acclimations, but under natural conditions food availability, along with its quantity and quality, differs seasonally. The number of seeds and its proportion in the diet increases towards winter (Fietz et al. 2005; Sailer and Fietz 2009), thereby increasing the availability of natural polyunsaturated fatty acids (PUFA). On the one hand, many studies showed that diets rich in PUFAs affect the energy expenditure of heterothermic animals by lowering their minimum *T*_b_ and metabolic rate during torpor (Geiser and Kenagy 1987; Frank 1992; Thorp et al. 1994; Florant 1998; Harlow and Frank 2001; Munro and Thomas 2004; Dark 2005; Geiser et al. 2007). On the other hand, diet rich in PUFAs may also be disadvantageous, as PUFAs are prone to auto-oxidation (Munro and Thomas 2004) what leads to lipid peroxidation. The production of toxic lipid peroxides may be detrimental to BAT, inhibiting hibernation (Frank and Storey 1995). Thus, animals would be protected against ROS, generated as a result of higher heat production and different diet, by endogenous increase in antioxidant capacity in winter.

## Conclusion

We found that oxidative status of heterothermic species changes both predictively and reactively (sensu Romero et al. 2009). It changes seasonally but it does not correlate with different winter phenotypes. We suggest that polymorphism of winter phenotype (responder—non-responder) persists in one population, because both phenotypes are beneficial under suitable environmental conditions (Kliman and Lynch 1992; Boratyński et al. 2016,2017; Przybylska et al. 2019a, b). Thus, phenotypic polymorphism can be viewed as an evidence of adaptive bet-hedging (Seger and Brockman 1987; Nevoux et al. 2010). On the one hand, responders partially reduce their expected fitness (because of shorter breeding season) in favour of enhanced survival of harsh winter (thanks to smaller body mass, regressed gonads and use of daily torpor). On the other hand, non-responders outcompete responders in less energy demanding environment. Thus, they can represent “go-for-broke” strategy which is usually unsuccessful but during mild winter permits successful breeding (Prendergast et al. 2001).

We propose that a seasonal increase in antioxidant defence is programmed endogenously to prevent oxidative stress in winter. Facultative non-shivering thermogenesis itself generates reactive oxygen species but its detrimental effects are presumably counterbalanced by upregulation of antioxidant levels. Thus, antioxidant defence is regulated over long (seasonal) and short (day-to-day, or even hour-to-hour) timescales protecting animals from oxidative stress and its detrimental consequences. In heterothermic species, regardless of responding or non-responding phenotype, winter is related to higher energy expenditure during periods of activity, and in responders, also to the use of torpor. Both situations, daily activity in the cold and returning to normothermia after torpor episodes, require a great increase in metabolic rate and thus increase ROS production. Endogenously programmed upregulation of antioxidant potential would protect animals against possible oxidative stress. This supports our hypothesis and suggests that evolution of different winter phenotypes was paralleled by the development of protective mechanisms allowing for their maintenance in stochastic environments.
